# The crystal structure of the human smacovirus 1 Rep domain

**DOI:** 10.1107/S2053230X23009536

**Published:** 2023-12-05

**Authors:** Lidia K. Limón, Ke Shi, Amy Dao, Jacob Rugloski, Kassidy J. Tompkins, Hideki Aihara, Wendy R. Gordon, Robert L. Evans

**Affiliations:** a Department of Biochemistry, Molecular Biology and Biophysics, University of Minnesota, Minneapolis, MN 55455, USA; Sungkyunkwan University School of Medicine, Republic of Korea

**Keywords:** HUH endonucleases, Rep domains, HUH-tags, ssDNA, bioconjugation, smacoviruses, crystal structure

## Abstract

Replication initiator proteins (Reps) from the HUH endonuclease family process specific single-stranded DNA sequences to initiate rolling-circle replication in viruses. Here, the first crystal structure of the apo state of a Rep domain from the smacovirus family is reported. The structure of the human smacovirus 1 Rep domain was obtained at 1.33 Å resolution and represents an expansion of the HUH endonuclease superfamily, allowing greater diversity in bioconjugation-tag applications.

## Abstract 2

The structure of the human smacovirus 1 Rep domain was obtained at 1.33 Å resolution. This new HUH endonuclease offers a new ssDNA-binding sequence specificity that will be exploited to increase orthogonality among Rep families.

## 1. Introduction

*Smacoviridae* is a family of small CRESS-DNA (circular Rep-encoding single-stranded DNA) viruses. These viruses have been found in the feces of multiple animals and are suspected to cause gastrointestinal disease in humans (Krupovic & Varsani, 2021 ▸; Li *et al.*, 2022 ▸). Indeed, CRESS-DNA viruses mainly infect eukaryotes. However, it was recently found that instead of direct infection of humans, smacoviruses may infect prokaryotes in the gut, making smacoviruses the smallest viruses to infect prokaryotes and functionally distinct from the majority of the family (Díez-Villaseñor & Rodriguez-Valera, 2019 ▸; Zhao *et al.*, 2019 ▸; Li *et al.*, 2022 ▸).

**Figure 1:**
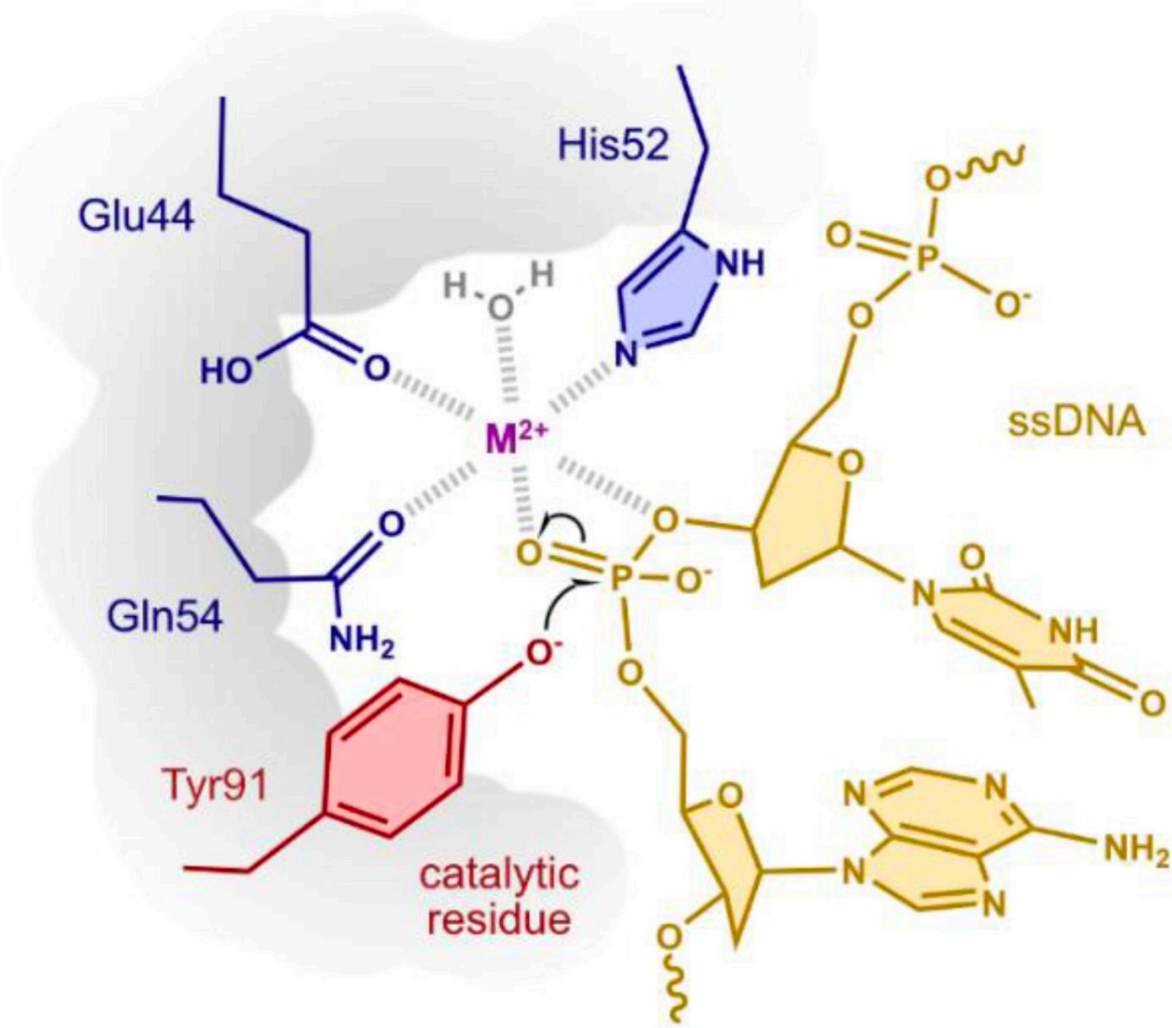
The catalytic activity of HUH endonucleases relies on the HUH/Q and tyrosine motifs to coordinate the nucleophilic attack on the DNA phosphate backbone (adapted from Tompkins *et al.*, 2021 ▸).

In addition to functional differences, there are putative structural differences in the replication initiator (Rep) domain in the HUH superfamily of enzymes responsible for processing single-stranded DNA (ssDNA) to replicate the genome during rolling-circle replication (Eisenberg *et al.*, 1977 ▸; Chandler *et al.*, 2013 ▸). Central to DNA processing of all HUH endonucleases is a structurally defined catalytic nickase domain that first recognizes a specific sequence/structure of DNA, nicks ssDNA at a ‘nic site’ to yield a sequestered 5′-end that remains covalently bound to the HUH endonuclease and a free 3′-OH that can be used as a primer for DNA replication, and finally facilitates a strand-transfer reaction to resolve the covalent intermediate (Fig. 1 ▸; Koonin, 1993 ▸; Ilyina & Koonin, 1992 ▸; Vega-Rocha *et al.*, 2007 ▸; Boer *et al.*, 2006 ▸; Chandler *et al.*, 2013 ▸; Lovendahl *et al.*, 2017 ▸). Named after a triad of residues, the HUH motif in the nickase domain is most often made up of two histidines separated by a bulky hydrophobic residue (U), but can also be histidine–U–glutamine. Several recent crystal structures have illustrated how viral Reps recognize and position ssDNA for cleavage (Luo *et al.*, 2018 ▸; Everett *et al.*, 2019 ▸; Tompkins *et al.*, 2021 ▸; Smiley *et al.*, 2023 ▸). Recent comparisons of CRESS-DNA Rep-domain protein sequences show that smacovirus Rep domains are both the smallest in size and the most divergent in sequence of the CRESS-DNA viral Reps (Tarasova & Khayat, 2022 ▸).

Finally, Rep domains from HUH endonucleases have been utilized as bioconjugation tags, termed HUH-tags, for applications that require covalent and specific protein–DNA bonds (Aird *et al.*, 2018 ▸; Sagredo *et al.*, 2016 ▸; Zdechlik *et al.*, 2020 ▸). Thus, structural information will guide their engineering to bind to desired DNA sequences (Tompkins *et al.*, 2021 ▸).

These interesting distinctions in function and domain composition suggest potential differences in structure and binding (*i.e.* bioconjugation) of the target DNA in smaco­viruses. As a first step towards understanding the structural basis for the function of the smacovirus Rep domain in prokaryote infection, we solved a 1.33 Å resolution crystal structure of a smacovirus Rep domain and made structural comparisons with other CRESS-DNA viral Reps.

## 2. Materials and methods

### 2.1. Protein production and purification

#### 2.1.1. Cloning

A codon-optimized gene block of the Rep-domain sequence from human smacovirus 1 (HSV1), accession No. AJE25845.1, was synthesized by Integrated DNA Technologies. An N-terminal His_6_-SUMO tag and 15 nucleotides homologous to the parent vector, pTD68, were included for cloning. The parent vector was linearized with the BamHI and XhoI restriction enzymes (New England Biolabs) and the gene block was ligated in using an In-Fusion HD Cloning Kit (Takara) as per the manufacturer’s protocol. The ligated plasmid was transformed into competent *Escherichia coli* Stellar cells and plated onto 100 µg ml^−1^ ampicillin plates. After overnight incubation at 37°C, colonies were chosen and DNA was purified with a Qiagen Miniprep kit. Confirmation of the purified plasmid was performed by Sanger sequencing (Genewiz). Protein-production details are provided in Table 1 ▸.

#### 2.1.2. Protein expression and purification

Verified plasmids were transformed into *E. coli* BL21(DE3) cells and cultured in 1 l Luria–Bertani (LB) broth with 100 µg ml^−1^ ampicillin at 37°C. The culture was induced at an OD_600_ of between 0.6 and 0.9 using 0.5 m*M* isopropyl β-d-1-thio­galactopyranoside and the cells were grown for 20 h at 18°C. The cells were harvested by centrifugation and the pellet was resuspended in lysis buffer (50 m*M* Tris pH 7.5, 250 m*M* NaCl). 1 m*M* EDTA and a protease-inhibitor tablet (Pierce, Thermo Fisher) were added to prevent metal binding and degradation, respectively. Lysis was performed via sonication at 1 min intervals at 4°C. The homogenous suspension was centrifuged at 24 000*g* for 25 min at 4°C. The supernatant was incubated for 1 h on a rotator with 2 ml HisPure Ni–NTA agarose beads (ThermoFisher) and equilibrated with wash buffer (50 m*M* Tris pH 7.5, 250 m*M* NaCl, 1 m*M* EDTA, 30 m*M* imidazole). The supernatant was loaded onto a gravity column and allowed to flow through. Protein-bound beads were washed with 25 ml wash buffer and the protein was eluted with 5 ml elution buffer (50 m*M* Tris pH 7.5, 250 m*M* NaCl, 1 m*M* EDTA, 250 m*M* imidazole). The eluted protein was dialyzed in 50 m*M* Tris pH 7.5, 150 m*M* NaCl, 1 m*M* EDTA. The His_6_-SUMO tag was cleaved with 5 µl ULP1 (1 U µl^−1^) overnight at 4°C and incubated with Ni–NTA agarose beads, and the flowthrough was collected. The protein-containing flowthrough was further purified using an Enrich SEC70 (Bio-Rad) size-exclusion chromatography column. Fractions containing the 16 kDa target protein were pooled and concentrated to 2.7 mg ml^−1^ using a spin concentrator (Amicon Ultra-15 Centrifugal Filter Unit, 3 kDa molecular-weight cutoff).

### 2.2. Crystallization

A protein solution containing a 10 bp DNA oligonucleotide sequence of the smacovirus origin of replication (AGTATTACGC) and Mn^2+^ was prepared in a 1:2:2 ratio. Drops consisting of 2 µl protein solution and 1 µl well solution were added to hanging-drop slides using the hanging-drop vapor-diffusion method. The well solution was composed of 0.1 *M* sodium acetate pH 5.0, 20% PEG 4000, 1 *M* guanidine–HCl. Upon crystal harvesting, 17% glycerol was added as a cryoprotectant. Crystallization details are listed in Table 2 ▸.

### 2.3. Data collection and processing

The data set was collected under cryoconditions on beamline 24-ID-C at the Advanced Photon Source (APS), Argonne National Laboratory using a Dectris EIGER2 16M pixel-array detector. The data set resulted in a 1.33 Å resolution model. Data-collection and processing details are provided in Table 3 ▸.

### 2.4. Structure solution and structure refinement

Molecular replacement with other viral Reps did not provide sufficient phasing information; therefore, a molecular-replacement search model was first generated by *AlphaFold*2 (Jumper *et al.*, 2021 ▸). The top generated model was then trimmed with *PyMOL* (version 2.0; Schrödinger) at the C-terminal end to remove short segments. The structure was solved with *Phaser* (McCoy *et al.*, 2007 ▸) using the trimmed *AlphaFold*2-predicted model and was refined with *Phenix* 1.17.1 (Liebschner *et al.*, 2019 ▸) and *Coot* (Emsley *et al.*, 2010 ▸). *MolProbity* (Chen *et al.*, 2010 ▸) was used for Ramachandran analysis. During refinement, it was determined that no ssDNA was bound to the structure. Structure solution and refinement statistics are listed in Table 4 ▸. The final model was deposited in the Research Collaboratory for Structural Bioinformatics Protein Data Bank as PDB entry 8fr5.

## 3. Results and discussion

### 3.1. Crystallization and structure determination

**Figure 2:**
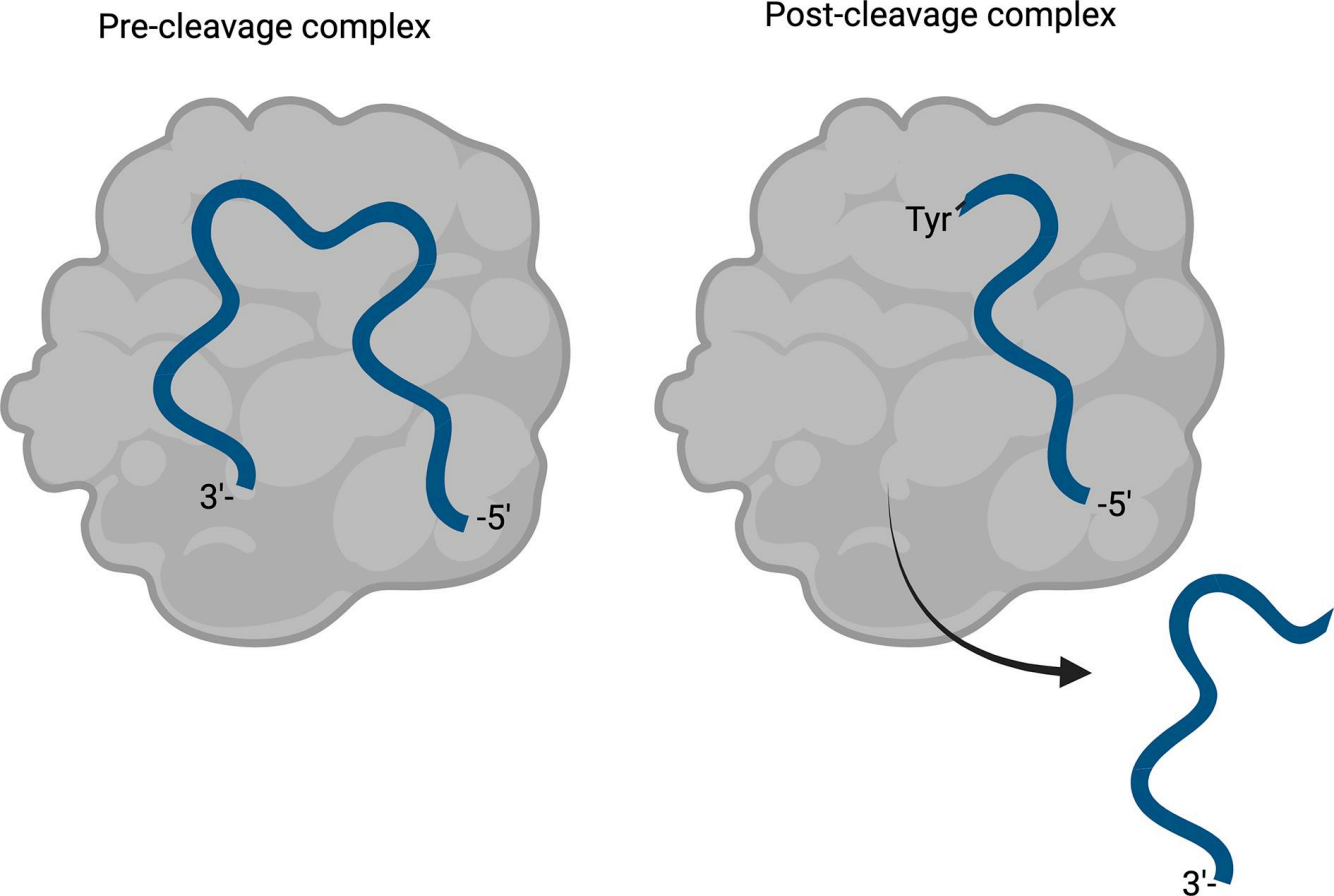
The coordination of the ssDNA by the Rep during the pre- and post-cleavage complexes. A mutation from tyrosine to phenylalanine does not allow the cleavage reaction to proceed but retains the ssDNA-binding ability of the Rep (Larkin *et al.*, 2005 ▸).

**Figure 3:**
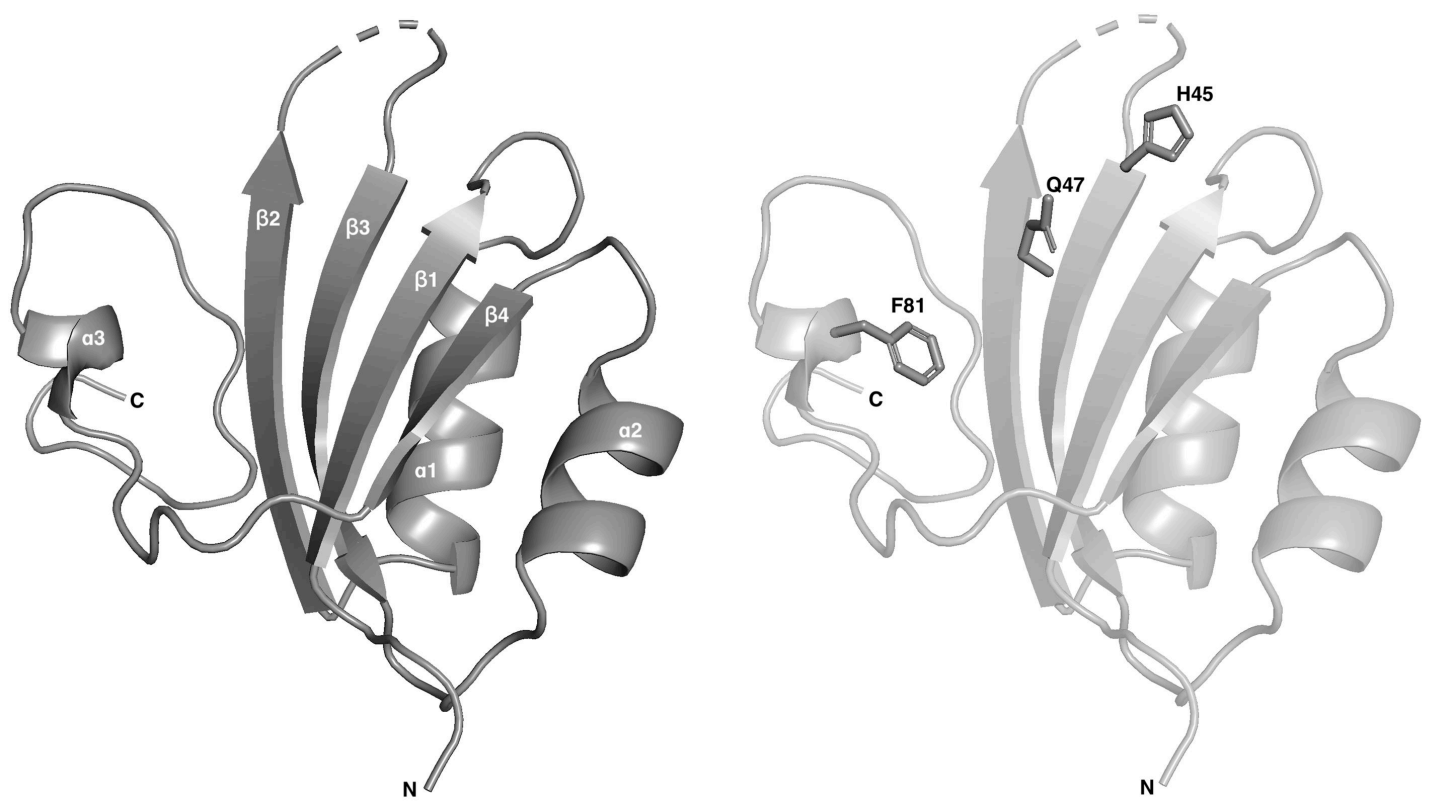
Ribbon model of HSV1 Rep showing the secondary-structure organization (left) consisting of β1, α1, β2, β3, α2, β4 and α3, and the orientation of the catalytic HUQ and tyrosine (Y81F in our model) motif (right). In our noncoordinated structure, the noncoordinating amino acid ‘U’ is oriented away from the binding site and is therefore not shown here.

To uncover structural differences compared with other ssDNA-bound HUH-tags, attempts to co-crystallize the ssDNA-bound protein were performed by mutating the catalytic tyrosine (Tyr81) to a phenylalanine. This allows the coordination of the ssDNA but not covalent linkage to the ssDNA (Larkin *et al.*, 2005 ▸). This is because the covalently linked ssDNA is cleaved and the orientation of the ssDNA is changed (see Fig. 2 ▸), which does not inform us as to the pre-cleavage coordination orientation. While attempts to obtain the bound/coordinated structure were unsuccessful, we did obtain an unbound structure of HSV1 Rep at 1.33 Å resolution (Fig. 3 ▸). The lack of 2*F*_o_ − *F*_c_ electron density supporting the absence of ssDNA bound to HSV1 Rep is illustrated in Supplementary Fig. S1. Protein crystals formed within days in many of the well conditions screened. The well condition that resulted in the largest crystals was 0.1 *M* sodium acetate pH 5.0, 20% PEG 4000, 1 *M* guanidine–HCl. The crystal belonged to space group *P*211. The unit-cell parameters were *a* = 31.16, *b* = 49.37, *c* = 31.38 Å, α = 90.00, β = 110.30, γ = 90.00°. There was one protein molecule in the asymmetric unit. The final values of *R*_work_ and *R*_free_ were 0.187 and 0.224, respectively.

### 3.2. Structure analysis

**Figure 4:**
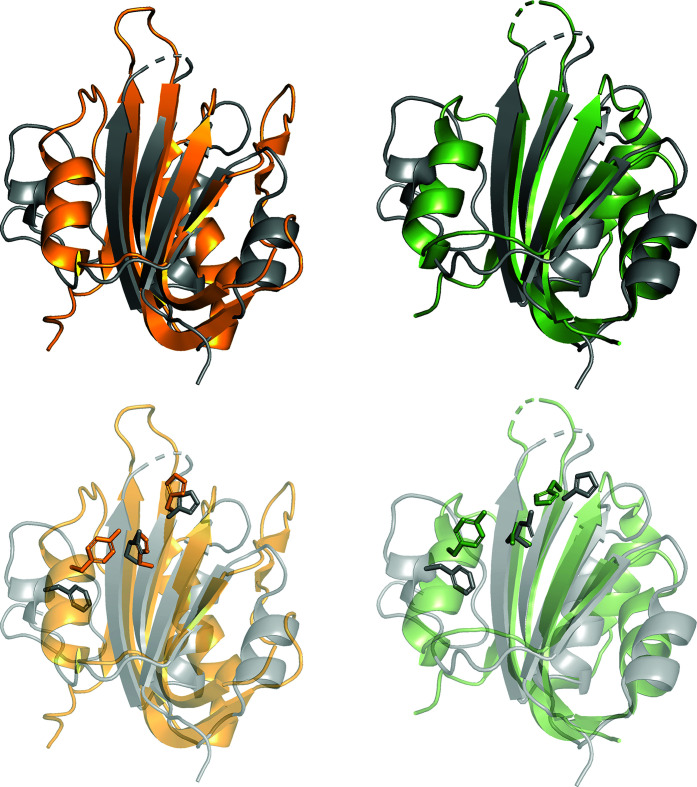
Structural alignment of HSV1 Rep (gray) with WDV Rep (orange, PDB entry 6q1m) on the left and PCV2 Rep (green, PDB entry 5xor) on the right. Superimpositions illustrate the structural conservation among the Reps (top) and the orientation of the catalytic HUH/Q and tyrosine residues (bottom). The r.m.s.d. value on superimposition of HSV1 and WDV is 2.4 Å and that for HSV1 and PCV2 is 3.3 Å.

**Figure 5:**
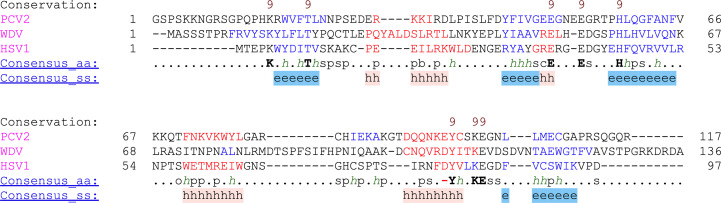
Structural and sequence comparison of HSV1 Rep with WDV Rep and PCV2 Rep using *PROMALS*3*D* (Pei *et al.*, 2008 ▸). β-Strands are shown in blue and α-helices in red. Consensus amino-acid symbols: conserved amino acids are shown as bold uppercase letters; *h*, hydrophobic; s, small; p, polar; c, charged; –, negatively charged.

Attempts were made to model the GEDG residues in the electron density adjacent to the HUH/Q motif (Chandler *et al.*, 2013 ▸) but were unsuccessful, indicating that the flexibility of the loop in this region is unrestrained, thus resulting in poor electron density. Modeling of ssDNA in the electron density adjacent to the catalytic domains, HUQ and tyrosine motifs for the bound/coordinated structure was also unsuccessful. The resulting unbound structure consists of β1, α1, β2, β3, α2, β4 and α3 secondary structures, with the β-sheets in an antiparallel layout (Fig. 3 ▸). The catalytically dead phenylalanine substituting for the reactive tyrosine residue resides within α3 and the coordinating histidine and glutamine residues reside within β3. The overall fold of Rep is highly conserved among families of Reps (Fig. 4 ▸). When a sequence and structure alignment was performed using *PROMALS*3*D* (Pei *et al.*, 2008 ▸), we found that the Rep from porcine virus 2 (PCV2; PDB entry 5xor) from the circovirus family is structurally closer to that from wheat dwarf virus (WDV; PDB entry 6q1m) from the geminivirus family than that from HSV1 (Fig. 5 ▸). This is in agreement with the r.m.s.d. values of the superimposed structures. On superimposition of HSV1 Rep with WDV Rep (PDB entry 6q1m) the r.m.s.d. is 2.4 Å, while that with PCV2 Rep (PDB entry 5xor) is 3.3 Å. This can be compared with the r.m.s.d. value of 0.96 Å between WDV Rep and PCV2 Rep. The difference may be due to the smaller protein size of HSV1 Rep, with fewer residues compared with WDV Rep and PCV2 Rep. HSV1 Rep is also structurally different from WDV Rep and PCV2 Rep in that α2 and α3 have shorter disordered loops connecting the α-helices to the β-sheets. Another difference among the families compared here is in the orientation of the HUH/Q and tyrosine residues in the catalytic motifs (Fig. 3 ▸), but this could also be explained by the absence of the divalent metal ion that is required to prime the active site for nucleophilic attack on the DNA substrate (Hickman *et al.*, 2002 ▸, 2004 ▸).

## Supplementary Material

PDB reference: human smacovirus 1 Rep domain, 8fr5

Supplementary FIgure. DOI: 10.1107/S2053230X23009536/ek5034sup1.pdf

## Figures and Tables

**Table 1:** Macromolecule-production information

Source organism	Human smacovirus
DNA source	Integrated DNA Technologies
Expression vector	pTD68
Expression host	*E. coli* BL21(DE3)
Complete amino-acid sequence	MTEPKWYDITVSKAKCPEEILRKWLDENGERYAYGRERGEDGYEHFQVRVVLRNPTSWETMREIWGNSGHCSPTSIRNFDYVLKEGDFVCSWIKVPD

**Table 2:** Crystallization

Method	Hanging drop
Plate type	24-well plate, Hampton Research
Temperature (K)	298
Protein concentration (mg ml^−1^)	2.7
Protein buffer solution	50 m*M* Tris pH 7.5, 50 m*M* NaCl
Volume and ratio of drop	3 µl, 2:1 (protein:reservoir)
Volume of reservoir (µl)	500

**Table 3:** Data collection and processing Values in parentheses are for the highest resolution shell.

X-ray source	Beamline 24-ID-C, APS
Wavelength (Å)	0.97910
Detector	EIGER2 16M
Exposure time (s)	0.20
Crystal-to-detector distance (mm)	170
Angle increment (°)	0.20
Resolution range (Å)	29.43–1.33 (1.38–1.33)
Space group	*P*211
*a*, *b*, *c* (Å)	31.16, 49.37, 31.38
α, β, γ (°)	90.00, 110.30, 90.00
Matthews coefficient (Å^3^ Da^−1^)	1.96
Solvent content (%)	37.33
Total reflections	71420 (5923)
Unique reflections	19692 (1733)
Multiplicity	3.6 (3.4)
Mosaicity (°)	0.12
Completeness (%)	91.30 (86.80)
Mean *I*/σ(*I*)	14.08
Wilson *B* factor (Å^2^)	19.40
*R*_merge_	0.042
*R*_meas_	0.049
*R*_p.i.m._	0.025
CC_1/2_	0.997

**Table 4:** Structure refinement Values in parentheses are for the highest resolution shell.

Reflections used in refinement	19612 (1679)
Reflections used for *R*_free_	1957 (168)
*R*_work_	0.187 (0.375)
*R*_free_	0.224 (0.414)
No. of non-H atoms	
Total	784
Macromolecules	736
Ligands	3
Solvent	45
No. of protein residues	92
R.m.s.d., bond lengths (Å)	0.005
R.m.s.d., angles (°)	0.77
Ramachandran favored (%)	100.0
Ramachandran allowed (%)	0
Ramachandran outliers (%)	0
