# Supplementary material for: The crystal structure of the human smacovirus 1 Rep domain
Source: Acta Crystallogr F Struct Biol Commun. 2023 Dec 5;79(Pt 12):295–300. doi: 10.1107/S2053230X23009536 (PMC10833120; doi:10.1107/S2053230X23009536)
Supplement: Supplementary file 1 [file f-79-00295-sup1.pdf]

Acta Cryst F
STRUCTURAL BIOLOGY COMMUNICATIONS

Volume 79 (2023)

Supporting information for article:

The crystal structure of the human smacovirus 1 Rep domain

Lidia K. Limón, Ke Shi, Amy Dao, Jacob Rugloski, Kassidy J. Tompkins, Hideki Aihara, Wendy R. Gordon and Robert L. Evans

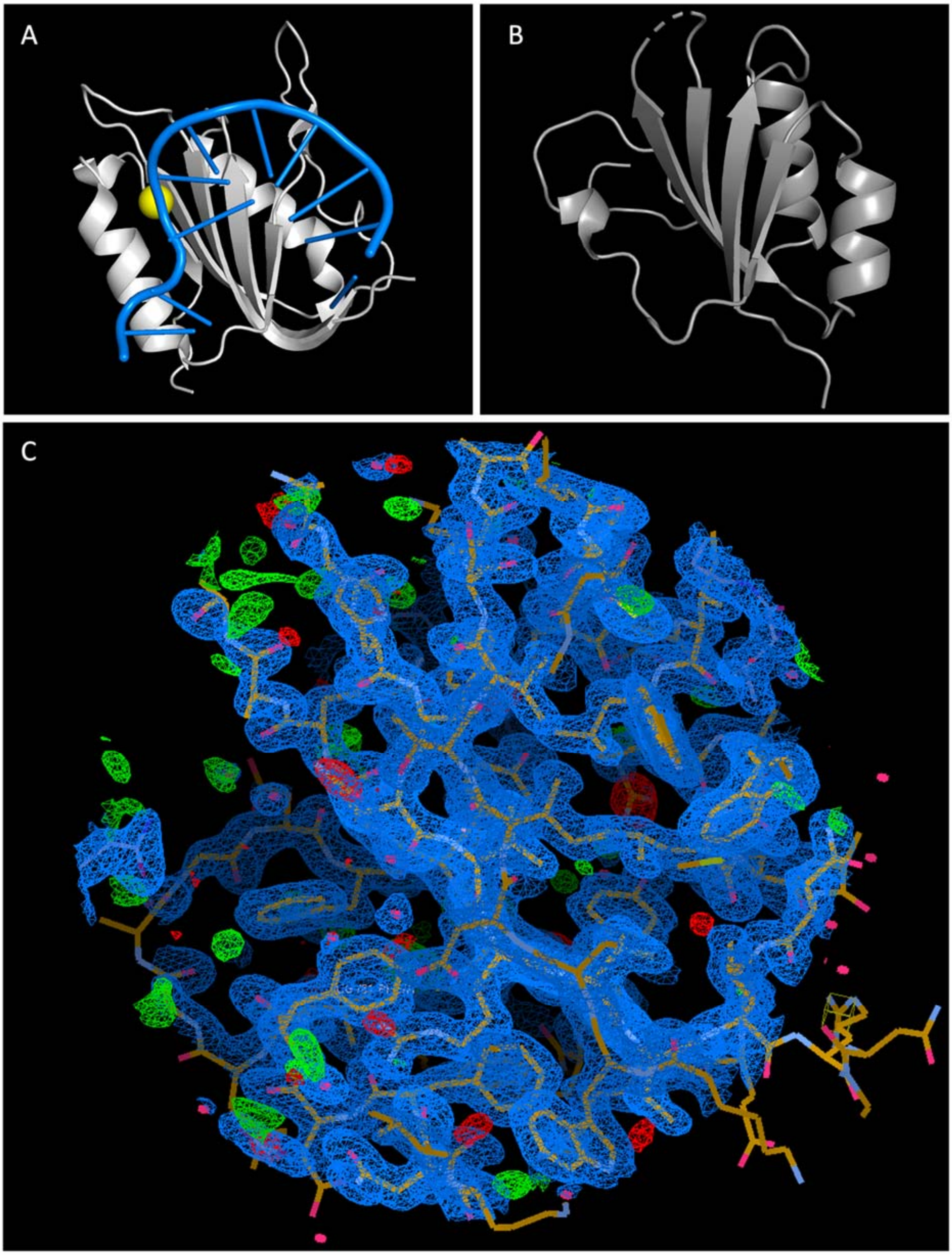

Figure S1 The molecules in panels A, B, and C are all being viewed from the same orientation. Panel A is the structure of the WDV Rep in the holo state with Mn^{2+} and ssDNA (PDB: 6WE0). Panel B shows the structure of the HSV1 Rep in the apo state (PDB: 8FR5). Panel C shows the 2Fo-Fc electron density (RMSD of 1.51) for the HSV1 Rep, supporting the absence of a Mn^{2+} and ssDNA.
